# Public Awareness and Attitude Towards Stroke in the Southern Region of Saudi Arabia: A Cross-Sectional Study

**DOI:** 10.7759/cureus.81910

**Published:** 2025-04-08

**Authors:** Abdullah I Aedh, Naif H Ali, Awam A Alsulaiman, Saleh Y Alyami, Ali I Alquraisha, Hussain M AlSulaiman, Hajar A Al Mustanyir, Raghad A Alsaiari, Hamza A Alalhareth, Zuhair M Almalki, Aeshah S Alqahtani

**Affiliations:** 1 Department of Internal Medicine, College of Medicine, Najran University, Najran City, SAU; 2 Department of Clinical Medicine, College of Medicine, Najran University, Najran City, SAU

**Keywords:** attitude, awareness, general population, saudi arabia, stroke

## Abstract

Introduction

Stroke is a leading cause of morbidity and mortality globally. Recognizing risk factors, identifying warning signs, and seeking timely medical intervention are crucial for preventing stroke and improving outcomes. This study aims to assess stroke awareness and attitudes among residents in southern Saudi Arabia and determine predictors of stroke knowledge.

Methods

A cross-sectional study was conducted among residents aged ≥18 years in the southern region of Saudi Arabia excluding individuals with a history of stroke and healthcare workers. A sample size of 576 participants was calculated. Data were collected conveniently via a self-administered online Arabic questionnaire that was adopted from the literature. The questionnaire assessed demographic characteristics, stroke knowledge, attitudes, and symptom responses. Data analysis was performed using multiple linear regression to identify predictors of awareness of stroke.

Results

A total of 510 participants (median age: 29 years, interquartile range: 24, 40) were included, with 288 (56%) being male. Stroke was identified as a brain disease by 287 (56%), and 371 (73%) recognized its preventability. Awareness of stroke symptoms ranged from 332 (65%) for sudden dizziness to 399 (78%) for speech difficulties. The most recognized risk factors were hypertension (443, 87%), smoking (395, 77%), and diabetes mellitus (342, 67%).

Better general awareness was associated with being married (β: 0.34, 95% CI: 0.07-0.61), divorced (β: 0.34, 95% CI: 0.07-0.61), or having a family history of stroke (β: 0.46, 95% CI: 0.22-0.70). Higher symptom awareness was linked to a family history of stroke (β: 0.92, 95% CI: 0.38-1.50) and earning 3000-10,000 RAS (β: 0.82, 95% CI: 0.11-1.50).

For risk factor awareness, family history of stroke (β: 0.96, 95% CI: 0.32-1.60) and earning 3000-10,000 RAS (β: 0.96, 95% CI: 0.12-1.80) were significant predictors. Awareness of stroke's consequence was higher among older participants (β: 0.02, 95% CI: 0.00-0.04) and those with a family history of stroke (β: 0.45, 95% CI: 0.09-0.81), while males (β: -0.32, 95% CI: -0.61 to -0.03) had lower scores.

Conclusion

The study highlights significant gaps in stroke awareness, particularly in recognizing symptoms with moderate overall knowledge of stroke risk factors. Targeted educational campaigns and community outreach programs for at-risk populations are essential to improving stroke awareness and ensuring timely medical intervention.

## Introduction

Stroke is a major cause of morbidity and mortality among the general population [1]. Its global impact highlights the need for a strong approach to reducing stroke risk [2]. Raising awareness and educating people about stroke is key to its prevention and management [3], which includes understanding risk factors, recognizing warning signs, and taking quick action when a stroke occurs [4]. Traditionally, prevention efforts have focused on increasing public knowledge, especially among high-risk groups [5].

In Riyadh, Saudi Arabia, the estimated stroke rate is about 43.8 per 100,000 people per year [6]. The average age of stroke onset in Gulf countries is relatively young, approximately 55 years, compared to developed countries [7] and compared to global figures, as a study on stroke characteristics in the Middle East and North Africa (MENA) region found the median age of stroke onset was 65 years, slightly younger than in non-MENA countries [8].

Increasing public awareness about stroke risk factors and warning signs can lead to faster responses and better treatment outcomes [9]. Community education programs are a key strategy in preventing strokes. These programs help assess people's knowledge about strokes and their risk factors [4, 10]. Educating the public not only improves their quality of life but also helps healthcare professionals manage stroke cases effectively in emergency settings [11]. Research suggests that 80% of stroke cases can be prevented through early action and precautions [12].

Studies among Lebanese adults show low awareness of stroke risk factors and the need to call emergency services quickly [13]. A study in Jordan found that stroke awareness is linked to education level and that factors like gender and socioeconomic status affect stroke outcomes [14]. In Riyadh, most people showed good knowledge of stroke risk factors and symptoms, but many did not understand the urgency of seeking emergency care [15]. Similarly, a study in Taif, Saudi Arabia, found that while people recognized stroke as a medical emergency, they struggled to identify its warning signs [16].

A study in Brazil found that 43.9% of people said they knew what a stroke was, 65% knew someone who had experienced it, 35% could name more than three risk factors, and 28.8% knew how to prevent it. Only 17.9% could list at least three stroke symptoms, 33.6% knew they should call emergency services, and just 3.1% said they would check when symptoms started [9]. However, studies have been conducted to assess stroke knowledge in the southern region of Saudi Arabia but they didn't target the general population [17-19]. Therefore, this study aims to assess stroke awareness and attitude among residents in the southern region of Saudi Arabia and to determine the predictors of awareness of stroke.

## Materials and methods

Study design

This was a cross-sectional study conducted among residents of the southern region of Saudi Arabia aged 18 years and above. Individuals with a history of stroke and healthcare workers were excluded to avoid bias from experience and prior knowledge respectively. The study covered three major regions in southern Saudi Arabia: Jazan, Najran, and Aseer.

Sample size

The sample size was calculated using the formula: n = z² (p(1-p))/d²

where d is the margin of error (5%), p is the expected proportion (50%), and z is 1.96 for a 95% confidence interval. The initial calculated sample size was 384 participants. To account for a design effect of 1.5, the minimum required sample size was adjusted to 576 participants.

Sampling technique and study instrument

Data was collected conveniently through Google Forms distributed through social media platforms using an online, Arabic self-administered questionnaire. The questionnaire was developed based on literature [14] and underwent face validity evaluation by a panel of neurologists. A pilot study involving 20 participants was conducted to assess the clarity and comprehensibility of the questionnaire. Feedback from the pilot study led to minor refinements, and responses from the pilot study were excluded from the final analysis.

The finalized questionnaire consisted of four sections; the first included demographic characteristics such as age, gender, occupation, education, marital status, income, and family history of stroke. The second assessed the awareness of stroke signs and symptoms, risk factors, consequences, and sources of information about stroke. The third evaluated the attitude and perception and beliefs about stroke, while the fourth assessed the actions and behaviors in response to patients with stroke symptoms.

Statistical analysis

Data was gathered in Microsoft Excel (Microsoft Corp., Redmond, WA, USA), cleaned, and analyzed using SPSS version 26 (IBM Corp., Armonk, NY, USA). The normality of continuous variables was assessed using histograms and Kolmogorov-Smirnov test. Descriptive statistics included frequencies and percentages for categorical variables and median with interquartile range (IQR) for continuous variables. For the knowledge score, correct answers were coded one, incorrect and insured answers were coded zero, and the total score and score for each subtype was calculated. Multiple linear regression analysis was performed to identify predictors of stroke knowledge. Multicollinearity was assessed using the variance inflation factor (VIF). Statistical significance was set at P <0.05.

Ethical considerations

This study has been approved by the scientific research ethics committee of King Khalid Hospital at Najran Health Cluster (IRB registration number with KACST, KSA:H-11-N-136). Informed consent was obtained electronically via a mandatory consent checkbox in Google Forms before participants could proceed with the survey. Participants were informed of their right to withdraw at any time. Data confidentiality was maintained by anonymizing responses, with no personally identifiable information collected.

## Results

The study included 510 participants with a median age of 29 years. More than half, 288 (56%), were males, 314 (62%) of participants primarily resided in the Najran region, and 249 (49%) were single. Most participants, 470 (92%), were Saudi nationals, and 428 (84%) had a university degree or higher. Regarding employment status, 318 (62%) were employed, 122 (24%) were students, and 70 (14%) were unemployed.

One hundred and fifty-seven (31%) earned less than 3000 RAS, 216 (42%) earned between 3000-10,000 RAS, and 137 (27%) earned more than 10,000 RAS. A family history of stroke was reported by 101 (20%) of participants (Table 1).

**Table 1:** Demographic characteristics of study participants The data has been represented as n (%) and Median (IQR).

Characteristic	N = 510^1^
Age	29 (24, 40)
Gender	
Female	222 (44%)
Male	288 (56%)
Residence	
Abha region	114 (22%)
Jazan region	82 (16%)
Najran region	314 (62%)
Marital status	
Single	249 (49%)
Married	218 (43%)
Divorced	29 (5.7%)
Widowed	14 (2.7%)
Nationality	
Non-Saudi	40 (7.8%)
Saudi	470 (92%)
Educational level	
Illiterate	3 (0.6%)
Primary school	6 (1.2%)
Secondary school	7 (1.4%)
High school	66 (13%)
University and above	428 (84%)
Occupation	
Unemployed	70 (14%)
Employed	318 (62%)
Student	122 (24%)
Income	
Less than 3000 RAS	157 (31%)
3000-10,000 RAS	216 (42%)
More than 10,000 RAS	137 (27%)
Family history of stroke	
Yes	101 (20%)
No	409 (80%)

Regarding stroke knowledge, 287 (56%) recognized stroke as a brain disease, while only 8 (1.6%) believed stroke was contagious. Seventy-three participants (14%) agreed that stroke is an elderly disease. Similarly, 62 (12%) believed stroke is hereditary, and 371 (73%) recognized stroke as a preventable disease. Regarding stroke symptom knowledge, 332 (56%) identified sudden dizziness as a symptom. A total of 347 (68%) recognized sudden blindness or double vision, while 350 (69%) identified sudden severe headaches. Sudden memory loss was recognized by 305 (60%), loss of consciousness or fainting by 332 (65%), sudden weakness or numbness in the limbs by 365 (72%), and difficulty speaking or understanding speech by 399 (78%). Regarding stroke consequences, 418 (82%) acknowledged that stroke can cause persistent disabilities, while 431 (85%) identified movement-related issues. Cognitive or memory problems were recognized by 412 (81%), visual problems by 374 (73%), and emotional or personality changes by 347 (68%) (Table 2).

**Table 2:** Knowledge of general information, symptoms and consequences of stroke among the study participants The data has been represented as n (%).

Characteristic	Yes	No	I do not know
General knowledge of stroke			
Stroke is a disease of the brain	287 (56%)	123 (24%)	100 (20%)
Stroke is contagious	8 (1.6%)	457 (90%)	45 (8.8%)
Stroke is an elderly person's disease	73 (14%)	353 (69%)	84 (16%)
Stroke is a hereditary disease	62 (12%)	306 (60%)	142 (28%)
Strokes can be prevented	371 (73%)	42 (8.2%)	97 (19%)
Knowledge of stroke symptoms			
Sudden dizziness	332 (56%)	69 (14%)	109 (21%)
Sudden blindness or double vision	347 (68%)	60 (12%)	103 (20%)
Sudden severe headache	350 (69%)	65 (13%)	95 (19%)
Sudden onset of memory loss	305 (60%)	78 (15%)	127 (25%)
Loss of consciousness or Fainting	332 (65%)	63 (12%)	115 (23%)
Sudden weakness/numbness tingling of arm/leg	365 (72%)	49 (9.6%)	96 (19%)
Sudden difficulty speaking or understanding speech	399 (78%)	40 (7.8%)	71 (14%)
Knowledge of the consequences of stroke			
Patients may encounter disabilities that persist long after the stroke is over	418 (82%)	92 (18%)	
Movement Functional problems (One-sided paralysis, Loss of ability to walk, tiredness, fatigue) after a stroke	431 (85%)	17 (3.3%)	62 (12%)
Cognitive/Memory problems after a stroke	412 (81%)	34 (6.7%)	64 (13%)
Visual problems (Loss of sight or blurred vision) after a stroke	374 (73%)	42 (8.2%)	94 (18%)
Emotional personality changes (Depression, anger, or mood changes) after a stroke	347 (68%)	43 (8.4%)	120 (24%)

High blood pressure was the most recognized risk factor, identified by 443 (87%), while smoking was recognized as a risk factor by 395 (77%).

Diabetes mellitus was identified as a risk factor by 342 (67%), whereas 376 (74%) acknowledged high cholesterol as a risk factor. Old age was associated with stroke by 344 (67%), while 397 (78%) recognized heart disease as a risk factor. Obesity was acknowledged by 383 (75%), whereas excessive alcohol consumption was identified as a risk factor by 425 (83%). Psychosocial stress was recognized as a risk factor by 442 (87%), while physical inactivity was identified as a contributing factor by 362 (71%) (Table 3).

**Table 3:** Knowledge of stroke risk factors among the study participants The data has been represented as n (%).

Characteristic	Yes	No
Knowledge of stroke risk factors		
High blood pressure	443 (87%)	67 (13%)
Smoking	395 (77%)	115 (23%)
Diabetes mellitus	342 (67%)	168 (33%)
High Cholesterol	376 (74%)	134 (26%)
Old age	344 (67%)	166 (33%)
Heart diseases	397 (78%)	113 (22%)
Obesity	383 (75%)	127 (25%)
Excessive alcohol	425 (83%)	85 (17%)
Psychosocial Stress	442 (87%)	68 (13%)
Physical inactivity	362 (71%)	148 (29%)

Regarding stroke symptoms, 60 (12%) could not identify any symptoms, while 84 (16.5%) recognized 1-3 symptoms, and 366 (71.6%) identified four or more. For general stroke knowledge, 28 (5.5%) had no correct knowledge, whereas 216 (43%) identified 1-2 key points, and 266 (52.4%) demonstrated knowledge of 3-5 aspects.

When evaluating stroke risk factors, 20 (3.9%) could not identify any, 35 (6.8%) recognized 1-3 factors, 77 (15.1%) identified 4-6 factors, and 378 (74%) were able to recognize 7-10 risk factors. For stroke consequences, 41 (8.0%) did not identify any, while 50 (9.8%) recognized 1-2 consequences and 419 (82.2%) identified 3-5 consequences (Table 4).

**Table 4:** Frequency of risk factors, early symptoms and consequences recognized by the study participants The data has been represented as n (%).

Characteristic	N = 510
Identified symptoms of stroke	
0	60 (12%)
1-3	84 (16.5%)
≥4	366 (71.6%)
Identified general information about stroke	
0	28 (5.5%)
1-2	216 (43%)
3-5	266 (52.4%)
Identified risks of stroke	
0	20 (3.9%)
1-3	35 (6.8%)
4-6	77 (15.1%)
7-10	378 (74%)
Identified consequences of stroke	
0	41 (8.0%)
1-2	50 (9.8%)
3-5	419 (82.2%)

The majority, 459 (90%), indicated that they would take a person having stroke symptoms directly to the hospital, while 25 (4.9%) would call a doctor. Twenty-four (4.7%) reported not knowing what to do, and only 2 (0.4%) stated they would wait for spontaneous recovery.

Regarding stroke education, 412 (81%) expressed interest in obtaining more information about stroke, while 98 (19%) were not interested. Additionally, 451 (88%) of respondents believed that family care plays a crucial role in the early recovery of stroke patients after discharge, whereas 59 (12%) did not share this belief (Table 5).

**Table 5:** Attitude towards stroke and responses to patients with signs of stroke The data has been represented as n (%).

Characteristic	N = 510
Response to a person with symptoms of stroke	
Take him/her directly to the hospital	459 (90%)
Call a doctor	25 (4.9%)
I don't know what to do	24 (4.7%)
Wait for spontaneous recovery	2 (0.4%)
Interested in having more information about stroke	
No	98 (19%)
Yes	412 (81%)
Family care is helpful for early recovery of stroke patients after discharge	
No	59 (12%)
Yes	451 (88%)

**Figure 1:**
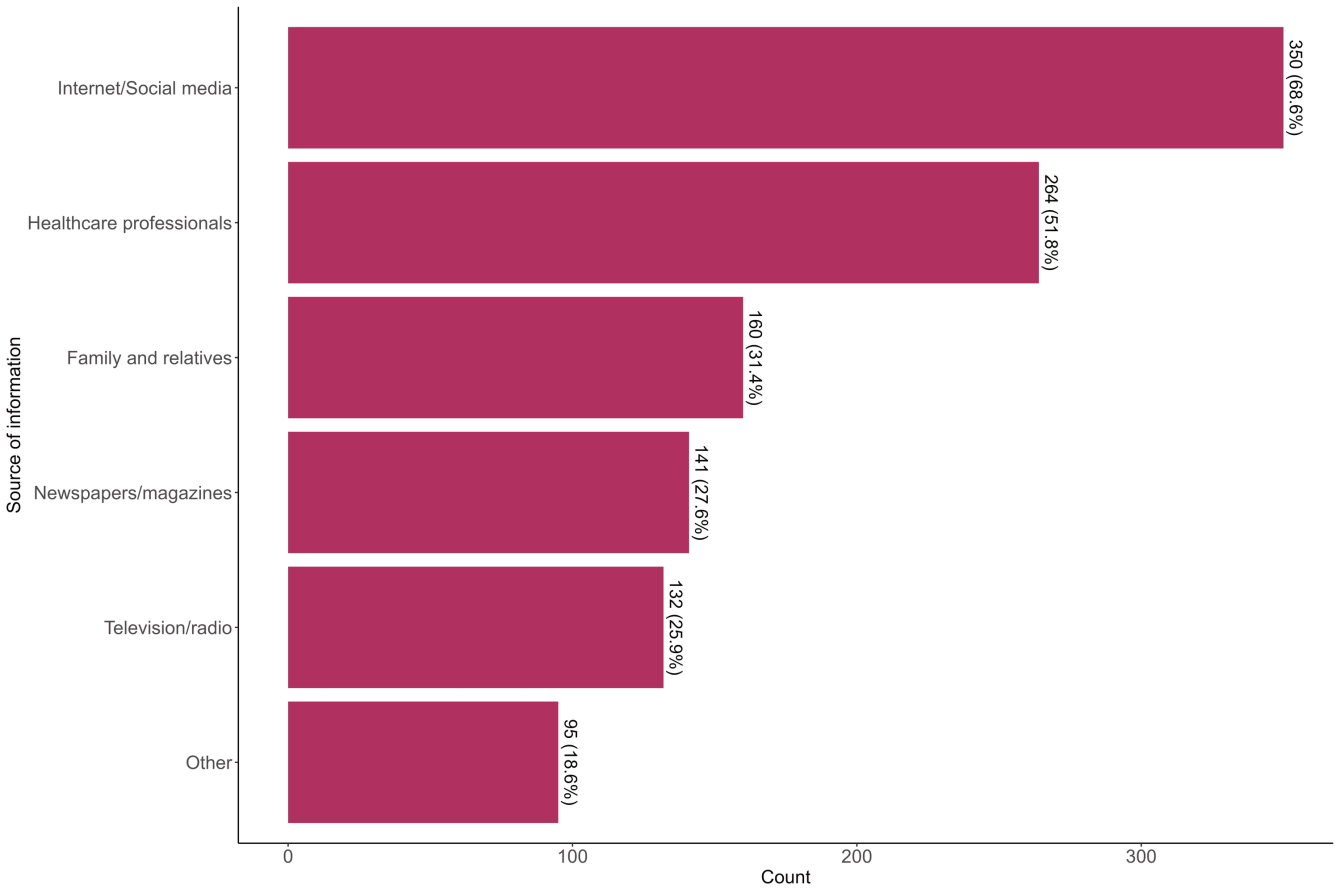
Source of information about stroke

The most cited sources of stroke information were Internet/social media which was cited by 350 (68.6%), 264 (51.8%) cited healthcare professionals and 160 (31.4%) cited family or relatives (Figure 1).

Regarding general knowledge of stroke, age showed no significant association with general knowledge (Beta: 0.00, 95% CI: -0.01, 0.01). Males scored slightly lower than females, but the difference was insignificant (Beta: -0.03, 95% CI: -0.22, 0.16). Participants from the Jazan region scored higher (Beta: 0.23, 95% CI: -0.06, 0.52), though the difference was insignificant. Married participants (Beta: 0.34, 95% CI: 0.07, 0.61), divorced participants (Beta: 0.34, 95% CI: 0.07, 0.61) and those with a family history of stroke (Beta: 0.46, 95% CI: 0.22, 0.70) showed significantly better knowledge. Higher-income levels were also associated with better knowledge, with significant differences for participants earning 3000-10,000 RAS (Beta: 0.49, 95% CI: 0.18, 0.81) and more than 10,000 RAS (Beta: 0.38, 95% CI: 0.03, 0.74).

For symptom knowledge, age, gender, and nationality were not significantly associated. Participants from Najran had significantly lower knowledge (Beta: -1.0, 95% CI: -1.6, -0.49). A family history of stroke was strongly associated with better symptom knowledge (Beta: 0.92, 95% CI: 0.38, 1.5). Participants earning 3000-10,000 RAS also showed better knowledge (Beta: 0.82, 95% CI: 0.11, 1.5).

Regarding risk factors, knowledge, family history of stroke (Beta: 0.96, 95% CI: 0.32, 1.6) and earners of 3000-10,000 RAS (Beta: 0.96, 95% CI: 0.12, 1.8) were significantly associated with better risk knowledge. Participants from Najran had significantly lower scores (Beta: -0.80, 95% CI: -1.4, -0.17).

For consequence knowledge, older age (Beta: 0.02, 95% CI: 0.00, 0.04) and family history of stroke (Beta: 0.45, 95% CI: 0.09, 0.81) were significantly associated with better consequence knowledge. Males had significantly lower consequence knowledge scores than females (Beta: -0.32, 95% CI: -0.61, -0.03). Participants from Najran showed lower consequence knowledge (Beta: -0.45, 95% CI: -0.82, -0.09) (Table 6).

**Table 6:** Determinants of knowledge of general information, symptoms, risk factors and consequences of stroke among the study participants 1*p<0.05; **p<0.01; ***p<0.001; beta = regression coefficient The data has been represented as beta value and 95% confidence interval. 2CI = Confidence Interval; P-value is significant at <0.05

	General knowledge		Symptom knowledge		Risk knowledge		Consequence knowledge	
Characteristic	Beta^1^	95% CI^2^	Beta^1^	95% CI^2^	Beta^1^	95% CI^2^	Beta^1^	95% CI^2^
Age	0.00	-0.01, 0.01	0.02	-0.01, 0.04	0.02	-0.02, 0.05	0.02*	0.00, 0.04
Gender								
Female	—	—	—	—	—	—	—	—
Male	-0.03	-0.22, 0.16	-0.07	-0.50, 0.36	0.50	-0.01, 1.0	-0.32*	-0.61, -0.03
Residence								
Abha region	—	—	—	—	—	—	—	—
Jazan region	0.23	-0.06, 0.52	0.39	-0.27, 1.0	0.48	-0.30, 1.3	0.26	-0.18, 0.70
Najran region	-0.01	-0.25, 0.23	-1.0***	-1.6, -0.49	-0.80*	-1.4, -0.17	-0.45*	-0.82, -0.09
Marital status								
Single	—	—	—	—	—	—	—	—
Divorced	0.49*	0.03, 0.95	0.57	-0.46, 1.6	0.71	-0.51, 1.9	0.33	-0.37, 1.0
Married	0.34*	0.07, 0.61	0.22	-0.39, 0.83	0.78*	0.06, 1.5	0.10	-0.30, 0.51
Widowed	0.35	-0.26, 0.96	0.97	-0.41, 2.3	1.5	-0.08, 3.2	0.48	-0.44, 1.4
Nationality								
Non-Saudi	—	—	—	—	—	—	—	—
Saudi	-0.04	-0.39, 0.32	-0.70	-1.5, 0.10	-0.45	-1.4, 0.49	-0.32	-0.85, 0.22
Educational level								
Illiterate	—	—	—	—	—	—	—	—
High school	-0.11	-1.3, 1.1	-0.47	-3.2, 2.2	0.65	-2.6, 3.8	0.36	-1.5, 2.2
Primary school	0.60	-0.83, 2.0	1.2	-2.1, 4.4	1.1	-2.7, 4.9	0.54	-1.6, 2.7
Secondary school	0.02	-1.4, 1.4	-0.50	-3.6, 2.6	1.3	-2.4, 5.1	1.1	-1.0, 3.2
University and above	0.24	-0.96, 1.4	-0.01	-2.7, 2.7	1.2	-2.0, 4.4	0.79	-1.0, 2.6
Occupation								
Unemployed	—	—	—	—	—	—	—	—
Employed	-0.11	-0.49, 0.26	-0.50	-1.3, 0.35	-0.04	-1.0, 0.96	-0.19	-0.75, 0.38
Student	0.33*	0.01, 0.65	0.23	-0.49, 0.95	0.74	-0.11, 1.6	0.29	-0.20, 0.77
Income								
Less than 3000 RAS	—	—	—	—	—	—	—	—
3000-10,000 RAS	0.49**	0.18, 0.81	0.82*	0.11, 1.5	0.96*	0.12, 1.8	0.44	-0.03, 0.92
More than 10,000 RAS	0.38*	0.03, 0.74	0.62	-0.19, 1.4	0.63	-0.32, 1.6	0.50	-0.04, 1.0
Family history of stroke								
No	—	—	—	—	—	—	—	—
Yes	0.46***	0.22, 0.70	0.92***	0.38, 1.5	0.96**	0.32, 1.6	0.45*	0.09, 0.81

## Discussion

This cross-sectional study aimed to assess the knowledge and attitude of the general population regarding stroke. It included 510 participants, from different geographical areas in Saudi Arabia including Najran, Abha, and Jazan. The majority were educated at the university level.

Assessing stroke knowledge among the general population in more detail revealed that 56% are aware that stroke is a primary brain disease, the same percentages were evident in socio-demographically and economically different geography, as 55.8% of Italian populations are aware that stroke affects the brain [20]. Ninety percent of our participants know that stroke is not contagious, and 12% think stroke is hereditary. In Riyadh (the capital city), higher percentages were aware of this aspect of stroke, as 91% acknowledged that stroke is non-infectious.

Family history of stroke is associated with better knowledge, these findings are consistent with national literature. Similarly to these findings, another study among the Saudi population found that a history of risk factors (hypertension, obesity, and hyperlipidemia) is associated with better overall stroke knowledge and better awareness regarding stroke symptoms. This is probably due to the fact that stroke is long-lasting, and family members are likely to be involved in the rehabilitation and care of stroke patients. Also, frequent hospital visits will make their contact with healthcare providers more and hence more likely to get more education on stroke recognition and care. The same applies to patients with risk factors who are likely to be educated by their frequent follow-up visits [21,22].

The majority agree that stroke is a preventable disease. Regarding their knowledge about stroke symptoms, sudden dizziness was identified by 56% of the participants. Sudden blindness and sudden severe headache were identified by 68% and 69%, respectively. The most commonly known symptoms among the general population were difficulty speaking or understanding speech and sudden weakness or numbness in the limbs identified by 78% and 72%. In a different region, a study conducted in Al-Ahsaa, Saudi Arabia, the most recognized symptom was sudden numbness or weakness identified by 80% [23].

However, participants showed a good level of knowledge regarding stroke risk factors. High blood pressure, smoking, alcohol, diabetes mellitus, obesity and high cholesterol were identified as possible risk factors. Nevertheless, 78% appreciated that cardiac disease could be a major risk factor while 85% pointed to psychosocial stress as a possible risk factor. The same set of risk factors was also reported by participants from the Balqarn region of Arabia in a study conducted by Al Ameer et al. [24]. However, in different publications among the Saudi population, physical inactivity was the most identified risk factor for stroke [22,25]. Upon assessing predictors of stroke-related knowledge, there was no statistically significant association with age, gender, and geography. A significant association was evident among groups with a family history of stroke and those with higher incomes between 3000-10,000 and more than 10,000 RAS. Compared to international figures, in Portugal elderly and patients with hypertension showed higher stroke-related knowledge. Same as the study among the population in Italy, the most identified risk factor was hypertension and the most recognized symptom was hemiparesis [20,26].

Compared with another Arabic population, the overall stroke knowledge was similarly found to be low among Egyptians, however, higher income, educational level, and having risk factors of stroke were associated with better knowledge [27]. Generally, the overall level of knowledge and participants' ability to recognize different stroke symptoms seem to differ according to the method of questioning. A review found that 25% to 72% of participants were able to identify stroke symptoms when asked open-ended questions, compared to 95% to 100% when asked close-ended questions. In our case, close-ended questions were used in data collection [28]. A large-scale study in Japan recommended the use of mass media and personal communication to enhance general population knowledge [29].

Assessing participants’ attitude, the majority indicated that they would take a person exhibiting stroke symptoms directly to the hospital. And more than two-thirds of the population expressed their interest to enhance their knowledge regarding stroke. A study in the Tabuk region of Saudi Arabia revealed that despite population uncertainty about stroke treatment, 85% had a positive attitude toward stroke [30].

Limitation

The study has several limitations; first, the use of convenience sampling via online distribution may introduce selection bias, as it primarily targets individuals with internet access, which affects the generalizability of the findings. Future studies could consider alternative sampling methods, such as stratified or random sampling, to improve representativeness. Secondly, the study did not assess attitude more extendedly, and did not standardize an overall score for both knowledge and attitude. However, this didn’t affect the outcome of this study as the comparison was mainly to the individual elements of knowledge and attitude.

## Conclusions

This study revealed a moderate knowledge of the general Saudi population regarding stroke symptoms and risk factors. A wider assessment of their source of information is needed to build baseline evidence for information dissemination in the future.

We recommend that more public educational campaigns be conducted to increase the population's overall knowledge of how to recognize stroke, how to deal with stroke patients acutely, and the possible first aid measurements that could be helpful in these situations. These programs should target, in the first place, the most at-risk population of stroke and their families. It could be delivered via television or social media as these are affordable, accessible and widely available. These campaigns can be governmentally funded and by healthcare workers.

## Appendices

**Table 7:** Questionnaire used to collect data

Questionnaire:								
Socio-demographic data								
Gender:	□ Male □ Female							
Age:								
Residence area:	□ Rural □ Urban							
Marital status:	□ Single □ Married							
Nationality:	□ Saudi □ Non-Saudi							
Educational level:	□ Illiterate □ Primary □ Secondary □University and above							
Employment status:	□ employed □ Unemployed □ Student							
Income level:	□ Low □ Medium □ High							
Family history of stroke:	□ Yes □ No							
Personal history of stroke:	□ Yes □ No							
Past Medical history:								
Has your doctor told you that you have one of the following health problems?								
Hypertension (High blood pressure):						□ Yes □ No		
Diabetes mellitus:						□ Yes □ No		
Dyslipidemia (High cholesterol level):						□ Yes □ No		
Arrhythmia (atrial fibrillation):						□ Yes □ No		
Kidney disease:						□ Yes □ No		
Peptic ulcer:						□ Yes □ No		
Depression:						□ Yes □ No		
Obesity/overweight:						□ Yes □ No		
Heart disease:						□ Yes □ No		
Have you smoked regularly for more than one year?						□ Yes □ No		
Familiarity with stroke								
Have you ever read/heard about a disease called stroke?				□ Yes □ No				
Knowledge about stroke:								
Is stroke a disease of the brain?					□ Yes □ No □ I don't know			
Is stroke contagious?					□ Yes □ No □ I don't know			
Is stroke an old person's disease?					□ Yes □ No □ I don't know			
Is stroke a hereditary disease?					□ Yes □ No □ I don't know			
Do you think strokes can be prevented?					□ Yes □ No □ I don't know			
How do you rate your knowledge about stroke?					□ Generally know □ Only name of the disease □ No knowledge			
Identification of Stroke symptoms								
What are the early symptoms of a stroke? Please choose all fitting symptoms from the following. If you think the following symptoms are related to the symptoms of stroke, please answer ‘Yes’; if you do not think so, answer “No”; If you are unsure, answer ‘I don’t know’.								
Sudden dizziness:							□Yes □No □ I don’t know	
Sudden blindness or double vision:							□Yes □No □ I don’t know	
Sudden severe headache:							□Yes □No □ I don’t know	
Sudden onset of memory loss:							□Yes □No □ I don’t know	
Loss of consciousness/Fainting:							□Yes □No □ I don’t know	
Sudden weakness/numbness/tingling of arm/leg:							□Yes □No □ I don’t know	
Sudden difficulty speaking or understanding speech:							□Yes □No □ I don’t know	
Risk factors of stroke								
Please select all the possible risk factors of stroke.								
Do you think “High blood pressure” is a risk factor of stroke?								□ Yes □ No □ I don't know
Do you think “Smoking” is a risk factor of stroke?								□ Yes □ No □ I don't know
Do you think “Diabetes” is a risk factor of stroke?								□ Yes □ No □ I don't know
Do you think “High Cholesterol” is a risk factor of stroke?								□ Yes □ No □ I don't know
Do you think “Old age” is a risk factor of stroke?								□ Yes □ No □ I don't know
Do you think “Heart diseases” is a risk factor of stroke?								□ Yes □ No □ I don't know
Do you think “Obesity” is a risk factor of stroke?								□ Yes □ No □ I don't know
Do you think “Excessive alcohol” is a risk factor of stroke?								□ Yes □ No □ I don't know
Do you think “Psychosocial Stress” is a risk factor of stroke?								□ Yes □ No □ I don't know
Do you think “Physical inactivity” is a risk factor of stroke?								□ Yes □ No □ I don't know
Source of information about stroke								
What are your information sources of knowledge about stroke? Please choose all the sources.								
□ Healthcare professionals (doctors, pharmacists, nurses,..) □ Had a previous stroke □ Family and relatives □ Internet/Social media □ Television/radio □ Newspapers/magazines □ Other								
Are you interested in having more information about strokes?								
□Yes □ No								
Consequences of stroke								
Do you know that patients may encounter disabilities that persist long after stroke is over?			□Yes □ No □ I don’t know					
What are the effects of stroke or the problems that a patient can experience after a stroke?								
Movement/Functional problems: One sided paralysis/Loss of ability to walk, tiredness/fatigue.		□Yes □ No □ I don’t know						
Cognitive/Memory problems: Loss of ability to speak, write, read, remember, or understand.		□Yes □ No □ I don’t know						
Visual problems: Loss of sight or blurred vision.		□Yes □ No □ I don’t know						
Emotional/personality changes: Depression, anger, mood changes.		□Yes □ No □ I don’t know						
Attitude toward stroke								
Do you think people who have strokes cannot lead a happy life?			□Yes □ No					
Do you think family care is helpful for early recovery of stroke patients after discharge?			□Yes □ No					
Respondent’s reaction to stroke symptoms:								
What will you do if you happen to witness a person with symptoms of stroke?								
□ Take him/her directly to the hospital □ Call a doctor □ Wait for spontaneous recovery □ I don't know what to do								
